# Microvascular decompression for hemifacial spasm involving the vertebral artery

**DOI:** 10.1007/s00701-021-05076-8

**Published:** 2021-12-06

**Authors:** Jing Wang, Yulong Chong, Chengrong Jiang, Yuxiang Dai, Weibang Liang, Lianshu Ding

**Affiliations:** 1 Department of Neurosurgery, Nanjing Drum Tower Hospital Clinical College of Nanjing Medical University, Nanjing, China; 2 Department of Neurosurgery, The Affiliated Huaian No.1 People’s Hospital of Nanjing Medical University, 6 West Beijing Road, Huaian, 223001 Jiangsu China

**Keywords:** Microvascular decompression, Hemifacial spasm, Vertebral artery, Neurovascular conflict

## Abstract

**Objective:**

Microvascular decompression (MVD) has become an accepted treatment modality for the vertebral artery (VA)–involved hemifacial spasm (HFS). The aim of this retrospective study was to evaluate clinical and surgical outcomes of HFS patients undergoing MVD and surgical and cranial nerve complications and investigate reasonable transposition procedures for two different anatomic variations of VA.

**Methods:**

Between January and December 2018, 109 patients underwent first MVD for HFS involving VA at Nanjing Drum Tower Hospital. Based on whether the VA could be moved ventrally at the lower cranial nerves (LCNs) level, patients were assigned to Group A (movable VA, *n* = 72) or B (unmovable VA, *n* = 37), and clinical and surgical outcomes and complications on the day of post-surgery and during follow-up were assessed. All patients were followed up ranging from 17 to 24 months with a mean follow-up period of 21 months.

**Results:**

After a mean follow-up of 21 months, the total cure rate significantly decreased in all patients compared to that achieved on the day of surgery, and Group A patients exhibited a higher cure rate versus Group B (93.1% vs. 75.7%, *P* = 0.015). Group B patients with unmovable VA revealed both higher incidence of surgical complications (45.9% vs. 15.3%, *P* = 0.001) and frequency of bilateral VA compression (27% vs. 8.3%, *P* = 0.009) versus Group A. No significant difference was observed in long-term cranial nerve complications.

**Conclusions:**

VA-involved HFS can benefit from MVD strategies after preoperative assessment of VA compression. HFS patients with movable VA may receive better long-term efficacy and fewer complications. A Teflon bridge wedged between the distal VA and medulla gives rise to adequate space for decompression surgery.

## Introduction

Hemifacial spasm (HFS) is a rare neurovascular movement disorder characterized by unilateral, irregular, and paroxysmal facial muscle contractions [3, 10, 16]. The most common cause is the vascular compression of the facial nerve at its root exit zone (REZ) in the brainstem. Microvascular decompression (MVD) surgery is one of several treatment concepts that has been proven most effective [11, 14]. When vertebral artery (VA) compression occurs and directly results in HFS, adequate mobilization of VA is a determinant for successful treatment and persistent efficacy of procedures [8]. Multiple approaches and various materials have been proposed to transpose the VA, such as stitching the VA to the dura using a bioglue-coated Teflon sling or aneurysm clip, whereas these processes of creating a sling require more complex manipulation, which may result in more frequent complications and higher damage. In the present study, through shifting the VA in the ventral direction by inserting the Teflon pieces into the space between the VA and brainstem at more points, we found it was relatively useful and simple to operate. However, there is not enough space for partial VA-involved HFS to move the VA ventrally. To our knowledge, effective treatment strategies for this condition are scarce. So, we retrospectively reviewed clinical data from 112 patients with VA-involved HFS and assessed the relationship between compression pattern and anatomic variations.

## Materials and methods

### Patients

This single-center study was conducted in Nanjing Drum Tower Hospital. Between January and December 2018, 112 patients underwent first MVD for VA-involved HFS, but three cases discontinued follow-up, so we ultimately achieved a follow-up rate of 97.3%. Magnetic resonance imaging (MRI) was performed preoperatively to evaluate vascular anomalies. Patients were assigned to Group A (movable VA, *n* = 72), whose axial T2-weighted MRI demonstrated adequate space between the VA and petrosal bone at LCN level; otherwise, patients were assigned to Group B (unmovable VA, *n* = 37). The superior border of the volume of the subarachnoid space between the brainstem and skull base was the internal auditory canal, the inferior border was the jugular foramen, and the lateral border was the auditory nerve and the lower cranial nerves. The volume was measured by 3D slicer software (3.6.3 version, USA). The average diameter of the ipsilateral VA was measured at 3-mm intervals, of which confluence was the origin.

### Surgical procedures

**Fig. 1:**
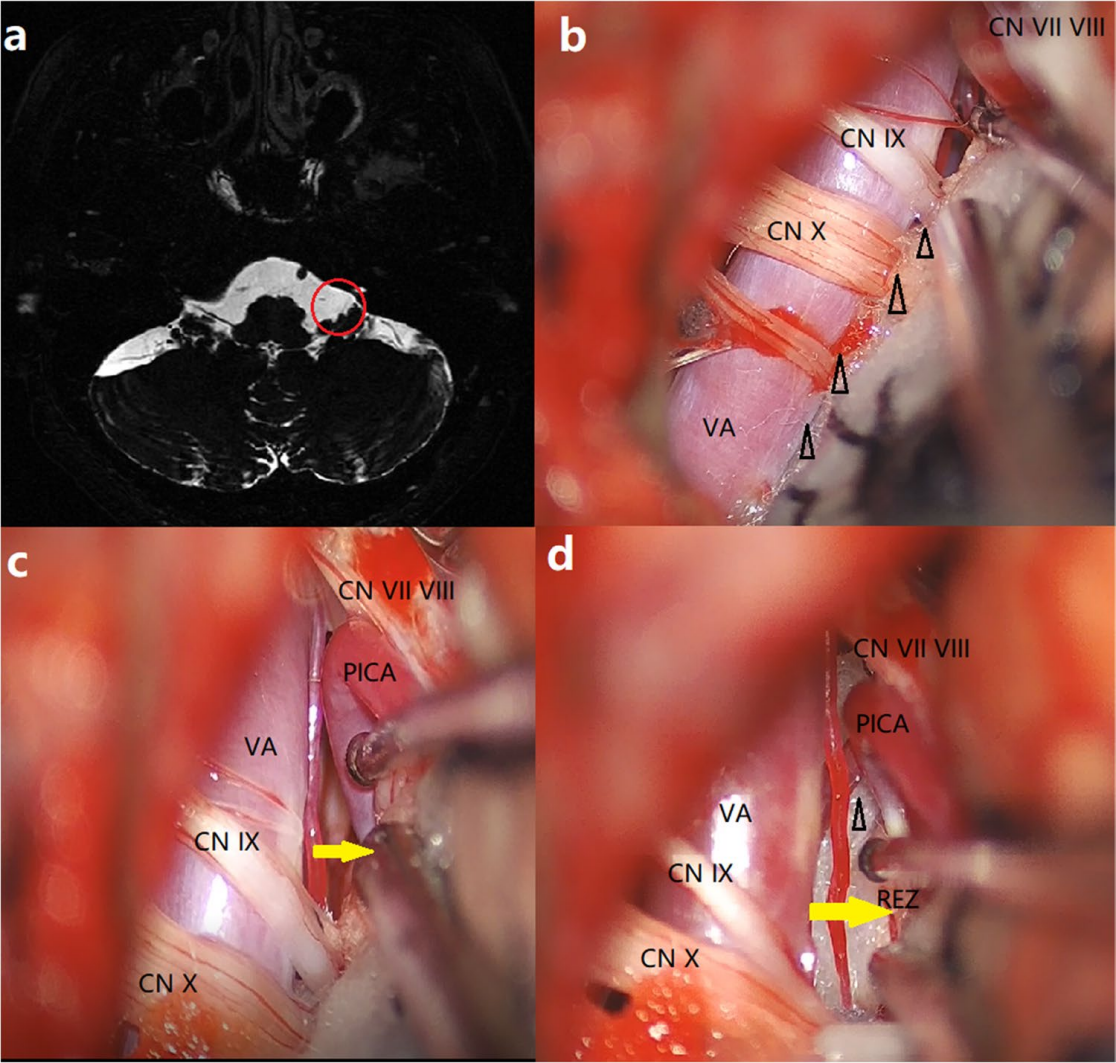
Preoperative MRI and intraoperative photos of Group A. **a** Preoperative axial T2-weighted MRI demonstrating adequate space (red circle) between the VA and petrosal bone at LCN level. **b** Teflon sponges (triangle) are placed between the VA and the medulla at the LCN level. **c** The space (yellow arrow) is expanded to expose the direct offender PICA. **d** Teflon sponges (triangle) are wedged between the distal VA and the medulla, thus creating a complete bridge for the VA. Yellow arrow shows the space between the REZ of the facial nerve and the VA. MRI magnetic resonance imaging, VA vertebral artery, LCN lower cranial nerve, PICA posterior inferior cerebellar artery, REZ root exit zone, CN cranial nerve

**Fig. 2:**
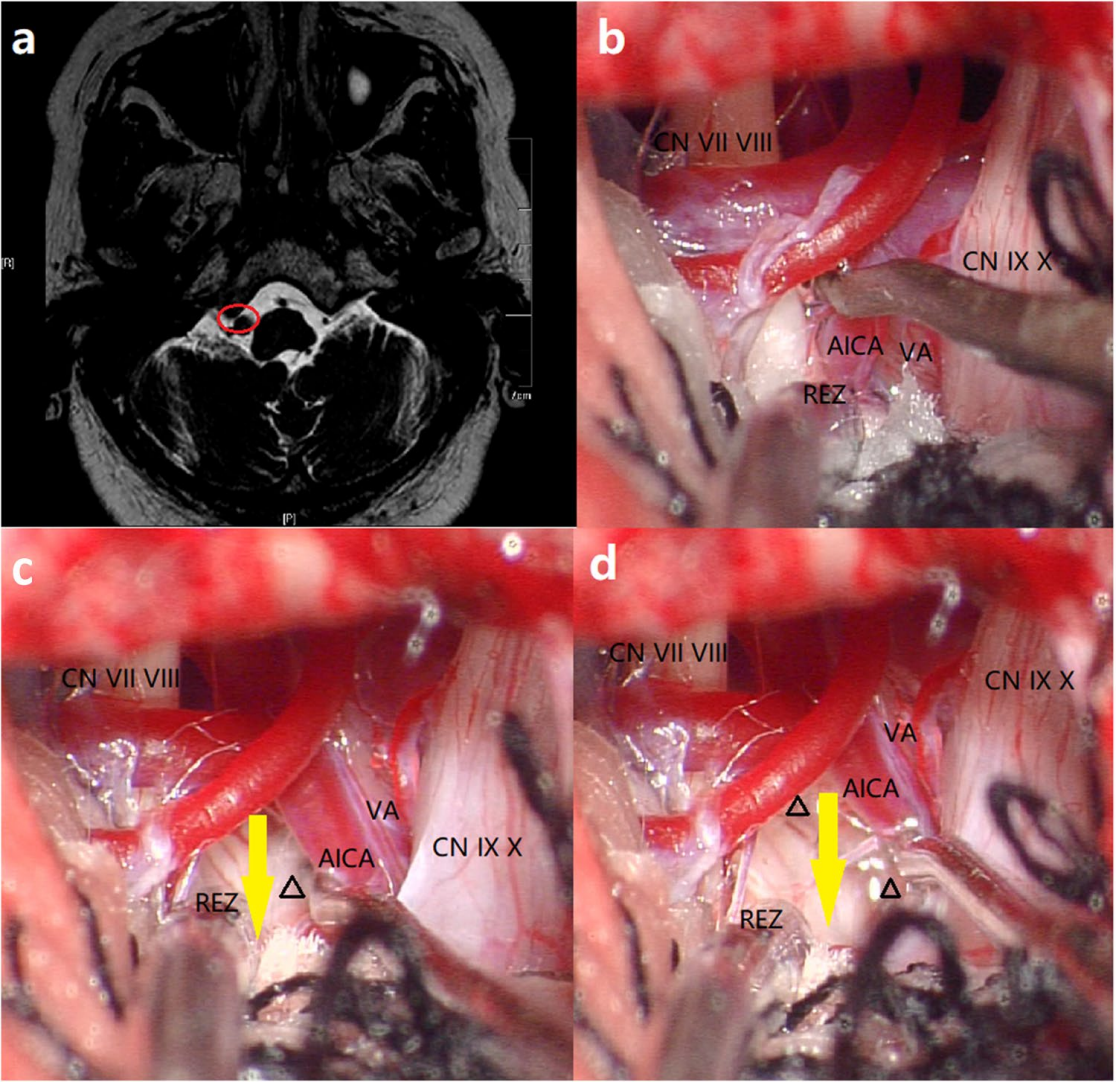
Preoperative MRI and intraoperative photos of Group B. **a** Preoperative axial T2-weighted MRI demonstrating no space (red circle) between the VA and petrosal bone at LCN level. **b** VA and AICA compress the REZ of CN VII. **c** Teflon sponges (triangle) are placed beyond the IXth cranial nerve level to expand the space (yellow arrow) between the REZ of the facial nerve and the offender. **d** Teflon sponges (triangle) are wedged between the distal VA and the medulla, thus creating a complete bridge for the VA. Yellow arrow shows the space between the REZ of the facial nerve and the offender. MRI magnetic resonance imaging, VA vertebral artery, LCN lower cranial nerve, AICA anterior inferior cerebellar artery, REZ root exit zone, CN cranial nerve

Prophylactic antibiotics was administered for the patients 0.5 h prior to surgery. Every MVD was performed using a suboccipital retrosigmoid approach with the patient in the lateral decubitus position under general anesthesia. The size of the craniotomy was enlarged inferiorly to expose the inferior aspect of the cerebellar hemisphere that was retracted from caudal to rostral to avoid mechanical pressure to the VIIIth cranial nerve after dura incision. The arachnoid membrane covering the lateral portions of the cerebellomedullary fissure at the XIth cranial nerve level was incised to remove the cerebrospinal fluid (CSF) and sharply dissected from the LCN level to the VIIth cranial nerve (facial nerve) level. If the VA could be moved ventrally at the LCN level, Teflon sponges were inserted into the junction between the VA and the medulla (Fig. 1). If VA transposition was impossible at the LCN level due to inadequate space, Teflon sponges should be placed beyond the IXth cranial nerve level to expand the space between the REZ of the facial nerve and the offender indirectly and were wedged between the distal VA and the medulla, thus creating a complete bridge for the VA (Fig. 2). The offender was isolated with a Teflon sponge to directly decompress the facial nerve until the lateral spread response (LSR) disappeared. If LSR was still present, the REZ was rechecked to ensure complete decompression. After finishing the MVD, the incisions were sutured layer by layer carefully to prevent CSF leakage.

LSR was a square-wave stimulation under 0.2 ms pulse width and 1 Hz frequency and recorded from orbicularis oculi muscle by stimulating the marginal mandibular branch of the facial nerve. LSR could be employed under a 20 mA stimulation and recorded during the operation continuously.

### Clinical follow-up

Various examinations were performed immediately after MVD and at the clinical follow-up to assess any development of neurological symptoms. Patients were required to participate in monthly follow-up visits or phone calls and followed up to 24 months. The clinical outcome score ranged from excellent (no HFS), good (partial relief or recurring spasm with improved patient satisfaction), to poor (minimal relief or no benefit). Excellent outcome was classified as cured. Excellent and good outcomes were judged as effective.

### Statistical analysis

All statistical analyses were performed using SPSS version 22.0 (IBM, Armonk, NY). Continuous variables were expressed as the mean ± standard deviation (SD), and categorical variables were expressed as percentages. Outcomes and the frequency of complications were compared between Group A and Group B using the independent *t*-test or chi-square test as appropriate. The significance level was set at *P* < 0.05.

## Results

### Patients, demographics, and symptoms

Finally, 109 patients were followed up 24 months after MVD. The clinical features of all the patients are described in Table 1. The mean age at onset was 50.1 ± 9.8 years (range 20–71 years), including 12/109 (11.0%) patients (range 20–39 years) and 97/109 (89%) patients (range 40–71 years). Left-sided HFS was common in both groups. The incidence of bilateral VA compression was significantly lower in Group A versus B (6 cases vs. 10 cases, *P* = 0.009). The diameter of the ipsilateral VA was significantly smaller in Group A, whereas the volume of the subarachnoid space between brainstem and skull base was larger than that of Group B. No significant differences were found in age, sex, side, or symptom duration between the two groups at baseline.

**Table 1:** Clinical characteristics and surgical results of the two groups

	Group A (*n* = 72)	Group B (*n* = 37)	*P* value
Gender (male/female)	33/39	17/20	0.991
Side (left/right)	57/15	25/12	0.184
Mean age (years)	55.7 ± 9.4	54.9 ± 9.3	0.670
Mean duration (years)	5.1 ± 4.7	5.6 ± 4.4	0.600
Volume of subarachnoid space (cm^3^)	6.4 ± 1.2	4.9 ± 0.7	0.027
Diameter of the VA (mm)	3.3 ± 0.6	4.3 ± 0.9	0.041
Offending vessels			
Bilateral VAs	6 (8.3%)	10 (27.0%)	0.009
VA + AICA	41	18	
VA + PICA	17	6	
VA + AICA + PICA	7	3	
VA	1	0	
Surgical outcomes			
Cured immediately	69 (95.8%)	33 (89.2%)	0.180
Long-term cured	67 (93.1%)	28 (75.7%)	0.015
Effective immediately	69 (95.8%)	33 (89.2%)	0.225
Long-term effective	71 (98.6%)	34 (91.9%)	0.112
Complications	11 (15.3%)	17 (45.9%)	0.001
Transient cranial nerve complications	8 (11.1%)	13 (35.1%)	0.003
Facial paralysis	3	9	
Hearing loss	4	2	
Hoarseness/dysphagia	0	2	
Tinnitus	1	0	
Others			
CSF leakage	1	0	
Hemorrhage	Remote site2	Remote site2	
	Surgical site0	Surgical site2	
Permanent cranial nerve complications	5 (6.9%)	4 (10.8%)	0.485
Facial paralysis	0	2	
Hearing loss	4	2	
Tinnitus	1	0	

### Clinical and surgical outcomes

During the follow-up, the long-term cure rate in Group A was significantly higher than that achieved in Group B (*P* = 0.015) (Table 1). There were no significant differences in the transient (95.8% vs. 89.2%, *P* = 0.225) and long-term (98.6% vs. 91.9%, *P* = 0.112) effective rates between them (Table 1). The overall effective rate at follow-up was 96.3% for all patients. Three (8.1%) cases in Group B were not ameliorated after surgery and were rated as poor.

During the follow-up, delayed resolution of residual HFS was observed in two (2.8%) cases of movable VA at 2 weeks and 3 months post-surgery, respectively. One (1.4%) case was not spasm-free until the end of follow-up, and four (5.6%) developed recurrent HFS with partial relief. Among patients with unmovable VA, delayed resolution occurred in one (2.7%) case at 3 months post-surgery. Six (16.2%) patients developed recurrent HFS, but symptoms were significantly reduced at 24 months follow-up.

### Complications

There was no mortality and infections in this study. Overall, patients with unmovable VA were more likely to develop postoperative complications and transient cranial nerve complications. Permanent complications were defined as the ones that continued until the last follow-up. There was no statistically significant difference in permanent cranial nerve complications between the two groups (*P* = 0.485).

## Discussion

The age of HFS at onset mostly ranges from 40 to 79 years [1], with a higher female prevalence found in most countries. In this study, VA-involved HFS was considered to have a higher female prevalence and the incidence rate was highest in those from 40 to 71 years of age, consistent with previous publications. Moreover, left-sided HFS was more common in VA-associated HFS. This may be explained by the fact that the left vertebral artery is often larger in diameter, resulting in asymmetric blood flow, making arterial angulation and tortuosity more frequent in the left VA. Thus, pulsatile compression occurs at the REZ of the facial nerve [2, 9].

The efficacy of MVD is often unsatisfactory in HFS associated with a large vessel, such as a VA offender, and patients generally have a fair surgical outcome [5, 12]. The anterior inferior cerebellar artery (AICA), posterior inferior cerebellar artery (PICA), or both AICA-PICA may act as culprit arteries that compress the REZ directly in VA-involved HFS. In our study, most of the patients were involved VA and other arteries. The offending vessel can always be found beneath the VA after VA transposition. VA transposition via various approaches, including transposition, insertion, and sling, has been explored [7, 13], some of which require complicated manipulations in such a narrow space, which is time-consuming and unsafe. For example, transposing an ectatic VA with a fenestrated aneurysm clip or anchoring with a thread to hold a dolichoectatic VA without injuring surrounding neural and vascular structures. It is also unfeasible to employ the same techniques for all VA-associated cases. In our study, Group B showed smaller subarachnoid space between brainstem and skull base and bigger diameter of the VA, that is, the difference between movable and unmovable VA was caused by VA size and size of the subarachnoid space, which increased the difficulty of the surgery. So, we strongly believe that surgical procedures in the deep and narrow spaces of the head and neck as VA-associated HFS must be as simple as possible to reduce surgical complications.

Placement of Teflon sponges between the VA and the medulla to create an adequate operative field for decompression is easy to operate and can produce a high success rate of MVD based on our experience of more than 400 cases over 5 years at our center. Given the high-risk operation to explore the REZ of the facial nerve directly, moving the VA and decompressing it at the LCN level largely reduce the risk of intraoperative complications. In our study, both groups showed a similar cure rate on the day of surgery, whereas patients with unmovable VA had a lower long-term cure rate compared to those with movable VA. So, we speculate that HFS patients with unmovable VA at baseline are more likely to have recurrent HFS post-surgery as unmovable VA means less anatomical space to circumvent compression even after surgical relief, which limits the efficacy of MVD against neurovascular conflict and the restoring force of the VA. Placement of Teflon sponges mechanically creates distance between the culprit artery and the REZ and offers comparable short-term relief in Group B patients that even incorporate more cases of bilateral VA compression versus Group A. For bilateral VA compression, the operative field is often smaller, resulting in inadequate decompression or new compression postoperatively [6, 15]. Caution should be exercised not to place Teflon bridges in the wrong site or overuse Teflon sponges. Generally, the Teflon bridge technique is reliable and relatively simple for VA-associated HFS.

Our experience of MVD in patients with unmovable VA, whose operative field is narrower, to reduce the incidence of transient cranial nerve complications and improve patient outcome at the follow-up can be summarized as follows. Preoperative evaluation of compression type on MRI is essential for better surgical decision-making or avoiding unnecessary surgical manipulations. Adequate arachnoid membrane dissection around the jugular foramen for the opening of the cerebellomedullary cistern allows extended LCNs exposure for observation during normal cerebellar retraction [4]. Moreover, gentle and skilled manipulations and the Teflon bridge technique give rise to complete decompression of the facial nerve root with minimized damage to the surrounding tissues.

## Conclusion

VA-involved HFS can benefit from MVD strategies after preoperative assessment of VA compression. HFS patients with movable VA may receive better long-term efficacy and fewer complications. Preoperative evaluation of VA compression type on MRI and complete decompression of the facial nerve root via Teflon bridges during minimal cerebellar retraction are the keys to successful MVD, and a Teflon bridge wedged between the distal VA and medulla gives rise to adequate space for decompression surgery.
